# TKTL1 is overexpressed in a large portion of non-small cell lung cancer specimens

**DOI:** 10.1186/1746-1596-3-35

**Published:** 2008-08-12

**Authors:** Holger Schultz, Daniel Kähler, Detlev Branscheid, Ekkehard Vollmer, Peter Zabel, Torsten Goldmann

**Affiliations:** 1 Research Center Borstel, Clinical and Experimental Pathology, Parkallee 3a, D-23845, Borstel, Germany; 2 Hospital Großhansdorf, Department of Thoracic Surgery, Wöhrendamm 80, D-22927, Großhansdorf, Germany; 3 Medical University Hospital III Lübeck/Department of Clinical Medicine, Research Center Borstel, Parkallee 35, D-23845 Borstel, Germany; 4 Ratzeburger Allee 160, D-23562 Lübeck, Germany

## Abstract

In several tumors the transketolase activity, controlled inter alia by enzymes of the pentose phosphate pathway which is an alternative, energy generating reaction-cascade to glycolysis, has been correlated with proliferation. The increase of thiamine-dependant transketolase enzyme reactions is induced especially through upregulated transketolase-like enzyme 1 (TKTL1)-activity; that shows TKTL1 to be a causative enzyme for tumors enhanced, anaerobic glucose degradation. We investigated TKTL1-expression in 88 human, formalin-fixed non-small cell lung cancer tissues and 24 carcinomas of the breast by immunohistochemical stainings applying a 0 to 3 staining-score system (3 = strongest expression). For means of validation we additionally stained 40 NSCLC fixed and paraffin-embedded utilizing the HOPE-technique; showing comparable results to the formalin-fixed, paraffin-embedded specimens (not shown). Potential correlations with age, sex, TNM-classification parameters and tumor grading as well as tumor transcription factor 1 (TTF1) and surfactant protein A (SPA) expression were investigated. 40.9% of the analyzed lung tumors expressed TKTL1 weakly (Score 1), 38.6% moderately (score 2) and 17.1% strongly (score 3). 3 tumors were diagnosed TKTL1-negative (3.4%; score 0). All Breast cancer specimen stainings were positive and scored 1: 32%; scored 2: 36%; scored 3: 32%. Alveolar macrophages and Alveolar Epithelial Cells Type II were also found to be TKTL1-positive.

None of the listed clinical parameters could be found to show a significant correlation to TKTL1 signal appearance.

Although we describe the expression of TKTL1 in lung cancers, we need to state that up till now there is no scientific indication for any treatment regimens based upon these findings.

## Findings

The ability of tumors to degrade glucose, even in the presence of oxygen, through the anaerobic transketolase-dependant pentose phosphate pathway has recently shown TKTL1 to particularly influence total transketolase activity and cell proliferation [1]. TKTL1 mRNA silencing (via small interfering anti-TKTL1-mRNA constructs) leads to inhibition of cell proliferation in colorectal cancer; protein overexpression and a significant correlation to Her2 overexpression was found in breast cancer cells where 89% expressed TKTL1 and 45% showed strong expression [2,3]. Gastric tumors and granulosa cell tumors of the ovary were also found to express high amounts of TKTL1 (36.9%; 81%) [4,5]. Further, with renal cancer another carcinoma displayed intensively elevated transketolase activity due to TKTL1-upregulation [6]. Proliferation-influencing activities of TKTL1 might play a role in a variety of cancers. Hence we investigated a capacious collective of 88 formalin-fixed non-small cell lung cancer (NSCLC)-tissues (39 adenocarcinomas; 49 squamous cell carcinomas) by immunohistochemistry to describe TKTL1 protein expression in human lung carcinomas. A collective of 24 breast cancer specimens was also included in the study (21 invasive-ductal; 1 tubular; 1 lobular; 1 mucinous carcinoma). Further, we challenged a potential correlation of TKTL1 to age, sex, TNM-classification and grading as well as TTF1 and SPA expression.

After lobectomy or pneumonectomy tumor tissues were immediately fixed with formalin and processed in the following over-night-procedure: formalin 1 h 30 min; following an alcohol series (70% 1 h 30 min; 80% 1 h; 96% 1 h for 2 times; 100% 1 h for 3 times; Histoclear for 1 h 2 times; subsequently paraplast is brought into the tissue at 60°C for 1 h 30 min; then another 2 h). Each step was followed by a 30 min. drop-off delay before the next step was started. After resection breast cancer specimens were processed in the same manner. Additionally we investigated 40 HOPE (Hepes Glutamic Acid Buffer Mediated Organic Solvent Protection Effect)-fixed NSCLC-tissue probes to validate the formalin fixation. After embedding in paraffin the blocks were shelved for 0–6 years within a tissue archive. For increased inter specimen comparability and even staining quality we utilized Tissue Microarrays (TMAs). These TMAs were produced from donor tissue blocks using an MTA1 (Manual Tissue Arrayer 1) device (Alphametrix, Germany), cut and mounted on microscope slides (Super Frost Plus, Langenbrink, Germany). For homogeneous high-throughput staining conditions the tissues were stained automatically (Autostainer 480, Medac, Germany).

The TMAs were deparaffinized and rehydrated by the following series at room temperature: 10 min. xylol incubation; 2 min. absolute alcohol (2 times); 2 min. 96% alcohol (2 times); 2 min. 90% alcohol; 2 min. 80% alcohol; 2 min. 70% alcohol; 2 min. aqua dest. (2 times). For antigen retrieval 30 min. citrate buffer cooking in was chosen. The primary antibody (anti-TKTL1; clone: JFC12T10; Zytomed Systems, Berlin) was incubated for 30 min. in a 1:100 dilution. We used an enzyme-polymersystem (ZytochemPlus HRP Polymer Kit, Zytomed Systems) for sensitive detection with permanent AEC (Zytomed Systems) as chromogen. Tissue arrays were counterstained by incubation in Mayer's haemalum for 5 minutes. Negative-controls were included in each staining series under omission of primary antibody.

**Figure 1:**
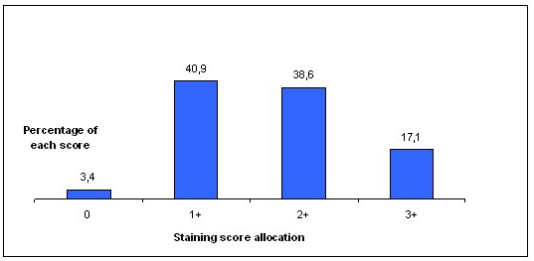
**Allocation of the stained lung cancer specimens to the four different staining scores**.

**Figure 2:**
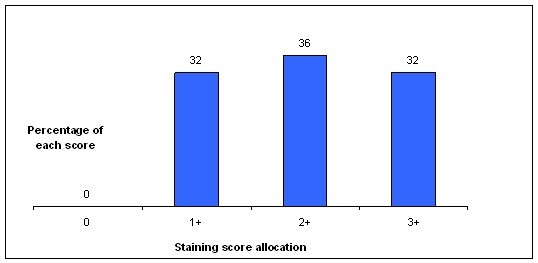
**Allocation of the stained breast cancer specimens to the four staining scores**.

The immunohistochemical stainings displayed elevated expression of TKTL1 in human lung cancer: 40.9% expressed TKTL1 weakly (score 1), 38.6% moderately (score 2), 17.1% strongly (score 3), and 3.4% of the tumors were TKTL1-negative (score 0; Figure 1). Breast cancer specimen stainings were scored 0: none; scored 1: 32%; scored 2: 36%; scored 3: 32% (Figure 2). In general, adenocarcinomas were more often strongly positive (score 3) than the squamous cell carcinomas (22.1% for adenocarcinomas and 12.2% for squamous cell carcinomas). Staining of the HOPE-fixed specimens showed comparable results; these results verify findings in the five cases of adenocarcinomas of the lung described by Langbein et al. [7].

**Figure 3:**
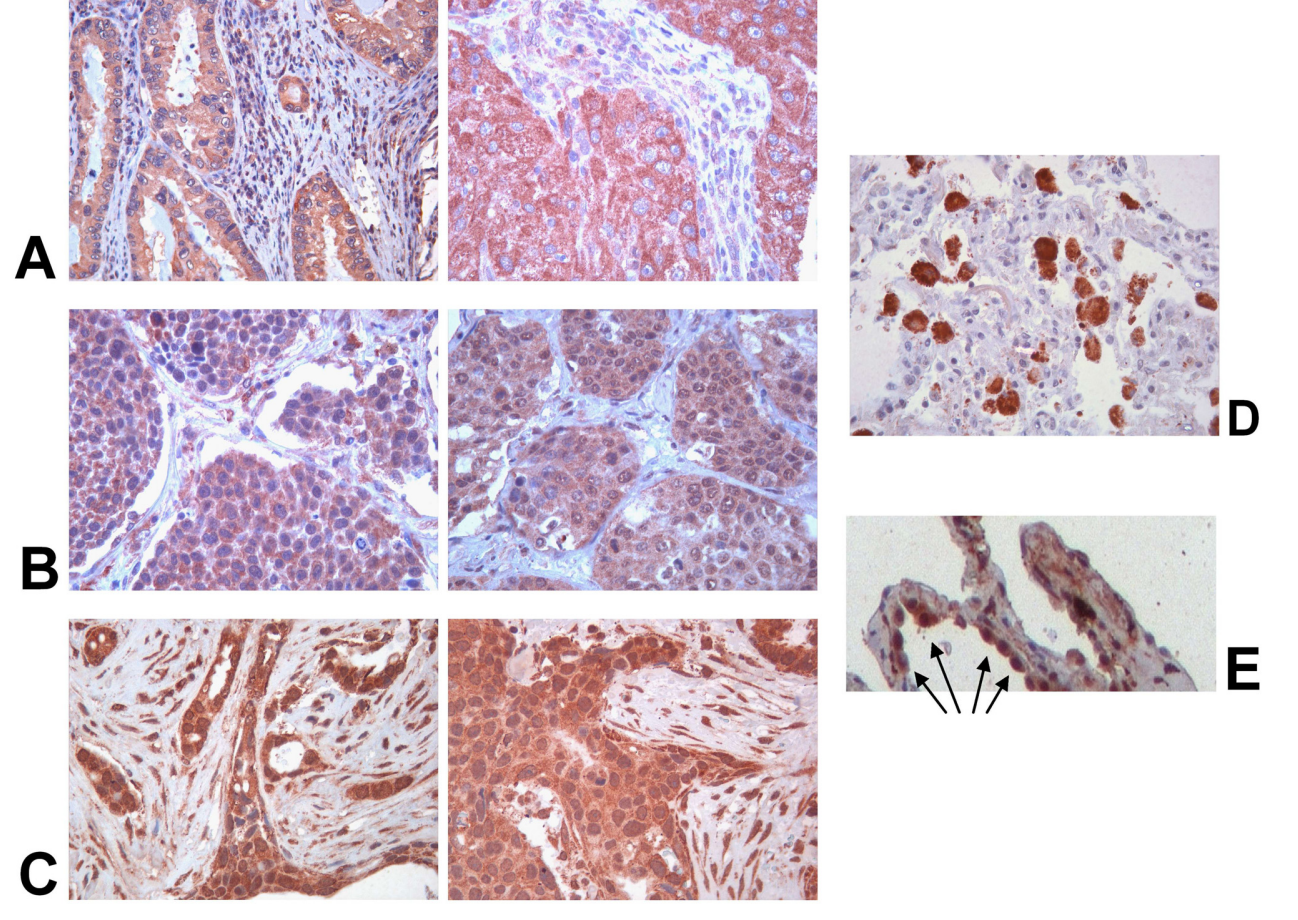
**Examples of TKTL1-stained A) adenocarcinomas; B) squamous cell carcinomas; C) breast cancer specimens; D) TKTL1-positive macrophages in the neighbourhood of an adenocarcinoma and E) TKTL1-positive AEC II cells in the neighbourhood of an adenocarcinoma (arrows)**.

Signals were recognized in the cytoplasm and occasionally in nuclei of tumor cells. There was a conspicuous amount of signal-containing alveolar macrophages and alveolar epithelial cells type II (AEC II cells), observed in the close neighborhood of the NSCLC (Figures 3D and 3E) as well as in completely healthy parts of lung tissue (not shown). Examples of stained tumor types, alveolar macrophages and AEC II cells are shown in figure 3A–E. No staining was found in negative controls. Comparison of TKTL1-expression to several clinical parameters revealed that there is no significant correlation between TKTL1-appearance and age, sex, TNM-classification parameters or tumor grading. Further no correlation could be defined concerning SPA and TTF1 expression.

A high variability of TKTL1-expression has been described in several tumor types. There is proven evidence that TKTL1-associated or -inducing, abnormal glucose degradation is increased in tumors [3-10]. But TKTL1 is also a key enzyme in the healthy organism whose activation-status influences the balance of anaerobic glucose- or oxygen-focussed bioenergy-obtainment. Natural appearance is proven through positive detected macrophages and AEC II cells (Figures 3D and 3E) as well as signals in healthy lung tissues. Therefore tests on cancer cells or cancer tissues alone are not capable to gain insights into the complex situation in patients subjected to diets or other treatments. We have shown that a large portion of NSCLC overexpresses TKTL1; moreover there was a significant expression in non malignant cells of the lungs. Although it is tempting to speculate of a potential therapeutic benefit by modulation of TKTL1-activity in the future, further studies are necessary to investigate the real amount of effects during TKTL1-targeted diets or therapies within the human organism.

## Competing interests

The authors declare that they have no competing interests.

## Authors' contributions

HS and DK drafted the manuscript and analyzed the sections. DB was responsible for the surgical part and clinical data. EV and PZ were responsible for the histopathological and the pneumological aspects. TG conducted the study together with EV and PZ and was involved in drafting the manuscript. All authors have read and approved the present manuscript.
